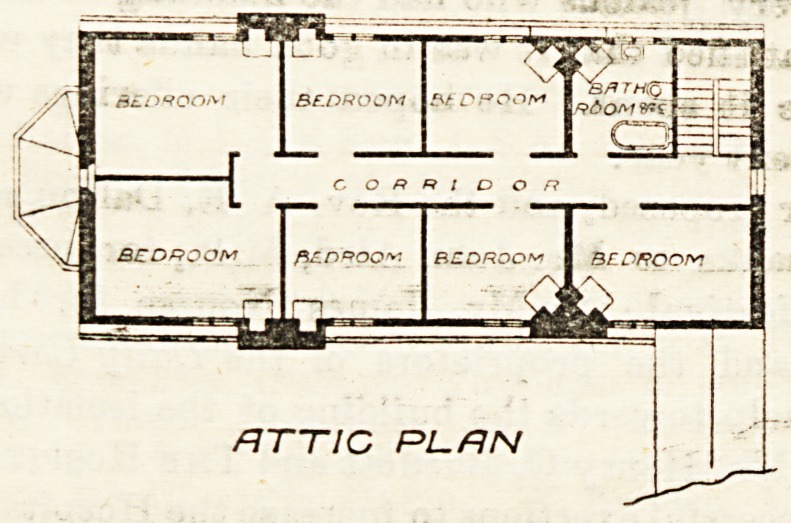# Hospital Construction

**Published:** 1896-03-28

**Authors:** 


### HOSPITAL CONSTRUCTION.

### THE ROYAL INFIRMARY, WIGAN.—EXTENSION OF THE GIDLOW WING.

Since the original infirmary for sixty beds was planned, additions have from time to time been made, nearly doubling the patients' accommodation, and

A, Extension to Gidlow Wing (Women's Ward); B, Special Wards and Nurses; C, Children's Wards; D, Women's Wards; E, Men's Wards; F, Accident Wards; G, Administration Block; H, Nurses' Dining Room; J, Future Isolation Block; K, Laundry; L, Mortuary.

making the building, as it appears on the block plan we publish, more irregular in shape than would be the case if it had in the first instance been designed for its full complement of beds. The latest addition, planned by Messrs. Worthington and Son, consists of a building of three storeys at the end of the Gidlow Ward, erected in memory of the lady after whom the ward was named, and by whom it was built. A new mortuary, with post-mortem, pathological, and waiting rooms in conjunction with it, has also been erected in an isolated position. The principal object of the addition to the main building has been to provide additional accommodation for nurses. On the ground floor, however, a ward for ten beds is arranged, with a bath-room and ward scullery opening out of the corridor by which it is approached. The sanitary appliances are in a separate block and properly disconnected from the ward. The ward is warmed by a central stove, and has in addition two open fireplaces in the angles. The arrangement of windows, doors, and beds is that usually adopted, and seems very good. The floor above is apportioned as a day-room for nurses and two small bed-rooms over the ward, a linen-room over the bath-room, and a library over the ward scullery. It is directly connected by a corridor lighted on both sides with the first floor in the Gidlow Ward building. A staircase leads to the top floor, which provides seven bed-rooms for nurses and a bath-room (with w.c. in the same apartment). Each bed-room has an open fireplace. With the single objectionable feature of placing the w.c. on this floor in the same room as the bath, the plans seem exceedingly well devised and simple, and the building should prove a most valuable addition to the institution.

## Figures and Tables

**Figure f1:**
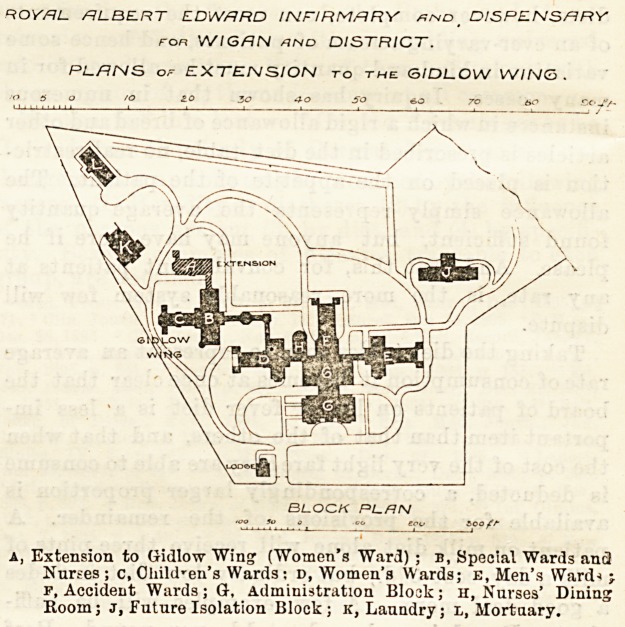


**Figure f2:**
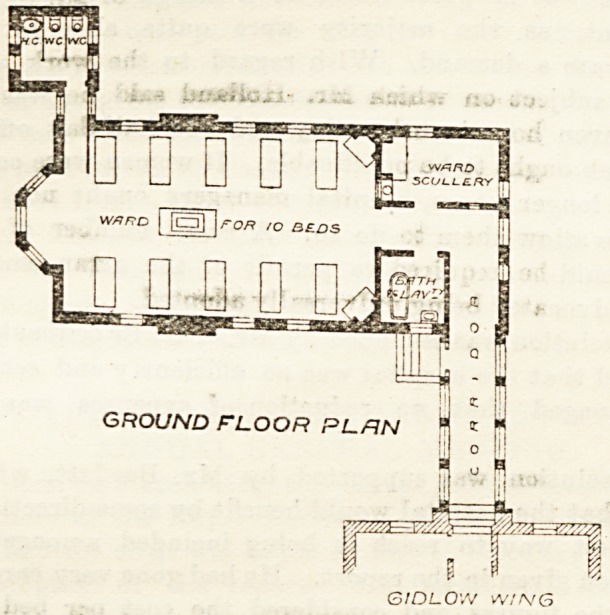


**Figure f3:**
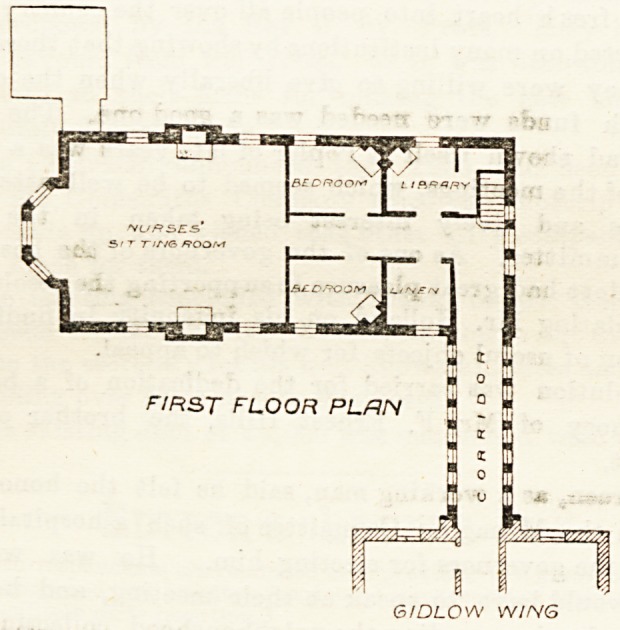


**Figure f4:**